# How key opinion leader characteristics influence women’s participation in soccer referee training

**DOI:** 10.3389/fpsyg.2026.1938303

**Published:** 2026-09-18

**Authors:** Kun-Feng Li, Chao Wang, Yi-Chi Zhang, Kai Hua

**Affiliations:** 1 Football College, Wuhan Sports University, Wuhan, China; 2 China Football Development Strategy Research Center, Wuhan Sports University, Wuhan, China

**Keywords:** female participation, KOL characteristics, perceived fit, perceived value, soccer referees, S-O-R theory

## Abstract

**Introduction:**

Drawing on the Stimulus–Organism–Response (SOR) framework, this study developed a theoretical model linking key opinion leader (KOL) characteristics,perceived value, perceived fit, participation intention, and participation behavior to examine how key opinion leaders influence women’s participation in soccer referee training.

**Methods:**

Data were collected through a questionnaire survey, yielding 744 valid responses. Structural equation modeling and bootstrap analysis were used to test the proposed hypotheses and mediating effects.

**Results:**

The results showed that KOL characteristics were positively associated with both perceived value and perceived fit, which were, in turn, positively associated with women’s participation intention. Participation intention further has a positive influence on actual participation behavior. However, the direct path of KOL characteristics, perceived value, and perceived fit on participation behavior are not significant, suggesting that women’s participation behavior is primarily shaped through the formation of participation intention. Bootstrap results further confirm that perceived value and perceived fit significantly mediate the relationship between KOL characteristics and participation intention, with 95% confidence intervals excluding zero.

**Discussion:**

This study extends the application of SOR theory to women’s sports participation and soccer refereeing, offering a new perspective on the psychological mechanisms underlying women’s engagement in referee training.The findings also provide practical implications for soccer governing bodies seeking to improve female referee training systems, strengthen the role of influential role models, and promote the sustainable development of women’s soccer refereeing.

## 1 Introduction

The rapid advancement of digital communication technologies has fundamentally reshaped the global soccer ecosystem. During the 2023 Fédération Internationale de Football Association (FIFA) Women’s World Cup, more than two billion people worldwide followed the tournament through television broadcasts, digital platforms, and social media channels (FIFA, 2023a; FIFA, 2024). This unprecedented level of audience engagement has not only enhanced the visibility of women’s soccer but has also transformed the production, dissemination, and consumption of sports-related information. Within this evolving communication environment, key opinion leaders, characterized by their professional expertise, social influence, and communication capabilities, have emerged as increasingly influential actors in promoting women’s soccer participation and shaping public perceptions of soccer refereeing. The career trajectory of French referee Stéphanie Frappart provides a compelling illustration of this phenomenon: She became the first woman to officiate matches in the Union of European Football Associations (UEFA) Super Cup (Union of European Football Associations [UEFA], 2019), the UEFA Champions League (Union of European Football Associations [UEFA], 2020), and the FIFA Men’s World Cup (FIFA, 2022). Her influence extends beyond sporting competition and into the broader public sphere; supported by extensive media exposure and digital communication networks, Frappart has become a highly visible role model who encourages women to consider refereeing as a viable professional pathway (Union of European Football Associations [UEFA], 2025). Furthermore, the expansion of social media platforms has facilitated the emergence of a new generation of referee-related key opinion leaders. For example, Italian assistant referee Giuliana Vigile has attracted more than 100,000 followers on Instagram, substantially increasing the visibility of the lower-division competitions in which she officiates Collectively, these cases suggest that key opinion leaders are assuming an increasingly prominent role in the development of football refereeing. Their growing visibility, credibility, and social influence have expanded their capacity to shape public perceptions of refereeing careers and may play an important role in encouraging women’s participation in referee training and professional development. Consequently, understanding the mechanisms through which key opinion leaders influence potential female referees has become an important research issue within contemporary sport management and communication research.

Although women’s soccer has experienced unprecedented global growth in recent years—FIFA reports indicate that approximately 16.6 million women and girls currently participate in organized soccer worldwide, a 24% increase from 2019 (FIFA, 2023b), and FIFA has established an ambitious target of reaching 60 million female players globally by 2027 (FIFA, 2025a)—the development of the female referee workforce has lagged considerably behind this rapid expansion. Despite substantial growth in female participation, women account for only 9% of registered soccer referees worldwide, and even in women’s leagues, female referees make up only 42%, with the proportion dropping sharply from 57% in top-tier leagues to 25% in second- and third-tier leagues (FIFA, 2025b). This imbalance is also evident at the national level. In England, for example, only 1,718 registered female referees are recorded, compared with 27,451 male referees (Grubb et al., 2023). Existing research suggests that women referees frequently encounter gender-based discrimination, verbal abuse, and harassment throughout their officiating careers (Gubby and Martin, 2025), they also experience inadequate institutional support, restricted career advancement opportunities (Silva-Santos et al., 2024), and unequal access to technological and professional development resources (Skirbekk, 2025), creating a structural imbalance between the rapid rise in participation and the career development of referees. Collectively, these challenges have created a structural mismatch between the rapid growth of women’s soccer and the sustainable development of the female referee workforce. Because referee training serves as the primary entry point into the profession, understanding the factors that encourage women to participate in referee training has become a critical issue for both sport management scholars and football governing bodies. Identifying the psychological and social mechanisms underlying training participation is therefore essential for expanding the pool of qualified female referees and supporting the long-term development of women’s football.

Existing scholarship on women sport officials has predominantly concentrated on structural and occupational challenges, including gender discrimination, workplace harassment, performance evaluation, leadership barriers, and career development constraints (Marshall et al., 2023; Skirbekk, 2024). Although these studies have substantially advanced understanding of the professional experiences of women referees, they have primarily adopted a problem-oriented perspective and paid comparatively limited attention to the antecedents of women’s entry into the refereeing profession. In particular, the psychological mechanisms underlying women’s participation intention in referee training and subsequently engage in training-related behaviors remain largely unexplored. This gap becomes even more salient within contemporary digital communication environments, where information acquisition, career exploration, and professional identity construction are increasingly influenced by key opinion leaders. Within the context of women’s soccer refereeing, key opinion leaders may function as influential external stimuli that shape how women perceive, evaluate, and interpret refereeing as a potential career pathway. Previous studies have consistently demonstrated that highly credible and professionally influential opinion leaders can shape attitudes, beliefs, and behavioral intentions through processes of social influence, identification, and role modeling (Kelman, 1958; Casaló et al., 2020). Nevertheless, behavioral responses rarely emerge directly from external stimuli. Instead, individuals typically process external information through a series of cognitive and psychological evaluations before behavioral intentions and actions are formed. A large body of empirical research has indicated that psychological variables at the perceptual level constitute the core pathway for explaining how external factors influence individual behavior. Among these psychological mechanisms, perceived value reflects individuals’ overall assessment of the benefits, utility, and attractiveness associated with a specific opportunity or profession (Lu H.-H. et al., 2024), whereas perceived fit represents the degree to which individuals perceive alignment between their personal characteristics, values, and the requirements of a particular role (Fang et al., 2024). Therefore, this study introduces perceived value and perceived fit as psychological variables.

Based on the foregoing discussion, this study seeks to advance current understanding of women’s participation in soccer referee training by examining the psychological mechanisms through which key opinion leader (KOL) characteristics influence training-related decision-making. Specifically, the study investigates whether and how KOL characteristics shape women’s intentions to participate in referee training and their subsequent participation behavior. Particular attention is given to the mediating roles of perceived value and perceived fit, two key psychological constructs that may explain how external social influences are translated into individual behavioral responses. Beyond examining their independent mediating effects, this study further explores whether perceived value and perceived fit operate as parallel psychological mechanisms linking KOL characteristics to training participation intention and behavior. By doing so, the study aims to provide a more nuanced explanation of how key opinion leaders facilitate women’s entry into the refereeing profession and to enrich the application of the Stimulus–Organism–Response framework within the context of women’s sport participation. The findings are expected to contribute to both the literature on women’s sport officiating and the broader understanding of how social influence processes shape participation in sport-related professions.

## 2 Theoretical framework and research hypotheses

### 2.1 Theoretical framework

#### 2.1.1 S-O-R theory

The Stimulus–Organism–Response (S-O-R) framework is one of the most widely adopted theoretical perspectives for explaining how external environmental stimuli influence individual behavioral outcomes through internal psychological processes. According to this framework, external stimuli affect individuals’ cognitive and affective states (organism), which subsequently shape behavioral responses. Rather than assuming a direct relationship between environmental factors and behavior, the S-O-R framework emphasizes the mediating role of internal psychological evaluations in the behavioral decision-making process.

Recent studies have extensively applied the S-O-R framework in digital communication and social media contexts. Research has demonstrated that external informational cues, such as content quality, interactivity, source credibility, and social influence, significantly affect individuals’ perceptions, attitudes, trust, and behavioral intentions (Islam and Rahman, 2017; Wang and Shahzad, 2024; Wu and Long, 2024). These findings suggest that behavioral responses are largely shaped by individuals’ cognitive interpretations of external information rather than by the stimuli themselves.

Within sport management and sport participation research, the S-O-R framework has also been increasingly employed to explain sport consumption, fan engagement, sport community participation, and career-related decision-making. Existing studies indicate that sport-related stimuli—including athlete images, sport influencer content, online interactions, and sport community experiences—can influence individuals’ professional perceptions, emotional attachment, identity construction, and sense of belonging, thereby affecting sport participation and career-related behavioral outcomes (Stegmann et al., 2024). Furthermore, research on women’s professional development has shown that female role models and opinion leaders can positively influence women’s career aspirations, professional attitudes, and participation motivations through social identification, role modeling, and value transmission processes (Steinke et al., 2024).

Accordingly, the S-O-R framework provides a particularly appropriate theoretical lens for examining women’s participation in soccer referee training. In the present study, KOL characteristics are conceptualized as external stimuli, as they represent important informational and social influence cues within contemporary digital communication environments. Perceived value and perceived fit are positioned as organism-level variables reflecting women’s cognitive evaluations and psychological responses to these stimuli. Participation intention and participation behavior constitute the response components of the framework. By integrating these constructs into a unified theoretical model, this study seeks to explain how KOL characteristics influence women’s participation in soccer referee training through underlying psychological mechanisms.

#### 2.1.2 Research on female soccer referees

Female soccer referees constitute a professional group of match officials responsible for enforcing the Laws of the Game and ensuring the fair, impartial, and orderly conduct of football competitions. Their responsibilities encompass a range of officiating roles, including referees, assistant referees, and video assistant referees (VARs), all of which are essential to maintaining the integrity and legitimacy of competitive football (The International Football Association Board [IFAB], 2024). Existing scholarship on female soccer referees has expanded considerably in recent years and can generally be categorized into four major research streams:

The first stream focuses on gender discrimination and male-dominated sporting cultures. Drawing primarily upon gender theory and the sociology of sport, these studies examine how female referees experience marginalization, stereotyping, and challenges to their professional authority within traditionally male-dominated football environments. Research suggests that female referees frequently face expectations to continuously demonstrate their competence and legitimacy, while their professional identities are often constrained by persistent gender stereotypes regarding women’s suitability for officiating football matches (Grubb et al., 2023); the second stream concerns career development and advancement barriers. Studies within this area investigate promotion pathways, access to training opportunities, mentoring systems, and career support mechanisms. Existing evidence indicates that structural gender inequalities continue to influence career progression within football officiating, limiting women’s access to professional development resources and leadership opportunities. Consequently, the establishment of formal mentoring structures and gender-inclusive career development systems has emerged as an important area of scholarly and practical concern (Şirin and Öz, 2024); the third stream focuses on the psychological experiences and well-being of female referees. Using predominantly qualitative approaches, these studies explore emotional labor, psychological stress, self-efficacy, professional identity, and resilience. Findings suggest that although female referees frequently encounter verbal abuse, challenges to their authority, and gender-based hostility during officiating activities, they often develop strong psychological resilience and professional commitment through continued engagement in the profession (Zanin et al., 2024); the fourth stream examines organizational support and institutional environments. Research within this area highlights the critical role of sport organizations, referee management systems, league structures, and gender equality policies in shaping women’s participation and retention within officiating. Increasing evidence suggests that the underrepresentation of female referees cannot be explained solely by individual-level factors but is also closely associated with organizational cultures, institutional barriers, and insufficient inclusivity within sport governance systems (Marshall et al., 2023); more recently, emerging studies have begun to explore how technological developments, public visibility, and role-model effects influence the professional experiences of female referees. Research indicates that technological innovations such as VAR systems may simultaneously create new professional opportunities and reinforce existing inequalities through uneven access to resources. Furthermore, growing public visibility has increased scholarly interest in how female referees influence broader perceptions of officiating careers and women’s participation in football-related professions (Skirbekk, 2025).

Despite these advances, several important limitations remain. First, existing research has largely focused on the challenges and experiences encountered by women already working as referees, while comparatively little attention has been devoted to understanding the factors that encourage women to enter the refereeing profession. Second, prior studies have predominantly emphasized structural constraints and organizational barriers, with limited examination of the psychological mechanisms through which women develop intentions to participate in referee training and subsequently engage in training-related behaviors. Third, although the visibility and influence of female referees have increased substantially, little research has explored how influential female role models and key opinion leaders shape women’s perceptions of refereeing careers and motivate participation in referee training.

Accordingly, a more comprehensive theoretical framework is needed to explain how external social influences are transformed into individual behavioral responses. By adopting the S-O-R perspective, the present study seeks to address this gap by examining how KOL characteristics influence women’s participation in soccer referee training through the mediating roles of perceived value and perceived fit.

### 2.2 Research hypotheses

#### 2.2.1 Characteristics of key opinion leaders

Key opinion leaders are individuals who possess substantial influence within a specific domain due to their expertise, credibility, visibility, and ability to shape public opinion. In contemporary digital communication environments, key opinion leaders play an increasingly important role in influencing how individuals acquire information, interpret opportunities, and make behavioral decisions. Drawing upon previous research, KOL characteristics are commonly reflected through dimensions such as professionalism, popularity, interactivity, homophily, and involvement. Collectively, these characteristics determine the extent to which audiences perceive key opinion leaders as credible, relatable, and influential sources of information. This study focuses on individuals who have visibility and professional influence in the field of soccer refereeing and who are capable of shaping potential participants’ perceptions and evaluations of referee training.

Participation intention refers to an individual’s subjective intention to engage in a particular activity and is generally regarded as an important antecedent of actual behavior. Within the context of soccer referee training, women often face uncertainty regarding professional requirements, career prospects, and personal suitability for the profession. Under such circumstances, key opinion leaders can serve as important informational and social reference points. Highly professional key opinion leaders provide valuable knowledge regarding refereeing pathways, training requirements, and career development opportunities, thereby reducing uncertainty and facilitating informed decision-making. At the same time, popular and highly visible key opinion leaders increase the social legitimacy and attractiveness of refereeing as a potential career pathway. Existing studies have consistently shown that source credibility, expertise, and social influence positively affect individuals’ attitudes, behavioral intentions, and participation intention in various activities (Crespo and Tille, 2024; Mabkhot et al., 2022).

In addition, the interactive and homophilous characteristics of key opinion leaders further strengthen their influence on participation intention. When audiences perceive similarities between themselves and opinion leaders, they are more likely to identify with the experiences and recommendations communicated by those leaders. Through interaction, communication, and experience sharing, key opinion leaders can enhance individuals’ confidence in their ability to engage in a particular activity and increase their motivation to participate (Rosara and Luthfia, 2020). Accordingly, KOL characteristics are expected to positively influence women’s participation intention in soccer referee training.

Beyond influencing participation intention, KOL characteristics may also affect actual participation behavior. Previous research suggests that opinion leaders can shape behavioral outcomes by providing behavioral models, demonstrating successful experiences, and reinforcing normative expectations (Shah et al., 2023; Chavda and Chauhan, 2024). Through continuous exposure to KOL-generated content, individuals may develop stronger professional aspirations and become more likely to translate intentions into concrete actions. Furthermore, the involvement and visibility of key opinion leaders can facilitate observational learning and role-model effects, encouraging individuals to emulate behaviors that are perceived as attainable and socially valued. Therefore, KOL characteristics may not only influence women’s participation intention in referee training but may also contribute directly to their training participation behavior.

Based on the above discussion, the following hypotheses are proposed:

*H1*: KOL characteristics positively influence female soccer referees’ participation intention in referee training.

*H2*: KOL characteristics positively influence female soccer referees’ participation behavior in referee training.

#### 2.2.2 The role of perceived value

Perceived value refers to an individual’s overall evaluation of the benefits obtained from a particular activity, profession, or opportunity relative to the costs required to engage in it (Zeithaml, 1988). As a cognitive assessment mechanism, perceived value plays a central role in shaping individual attitudes and behavioral decisions. Within the Stimulus–Organism–Response (S-O-R) framework, perceived value represents an organism-level cognitive response through which individuals interpret and evaluate external information. Consequently, perceived value is highly susceptible to the influence of environmental stimuli and information sources.

In the context of soccer referee training, key opinion leaders can significantly influence women’s perceptions of the functional value associated with the refereeing profession. Key opinion leaders possessing high levels of expertise and credibility are often viewed as reliable sources of information, enabling audiences to develop a clearer understanding of the knowledge, skills, and competencies required for successful officiating. Through the dissemination of professional knowledge, practical experiences, and career development information, key opinion leaders help reduce uncertainty regarding the profession and increase perceptions of its developmental and educational value (Munnukka et al., 2019). As a result, women may become more likely to perceive referee training as a meaningful opportunity for skill acquisition and professional growth.

Key opinion leaders may also enhance the emotional and social value associated with participation in referee training. Soccer refereeing is frequently perceived as a high-pressure profession characterized by public scrutiny and substantial psychological demands. Through experience sharing, emotional support, and role-model effects, key opinion leaders can reduce uncertainty and foster positive emotional connections with the profession. Previous research has demonstrated that emotionally engaging and credible communicators can strengthen audience attachment, identification, and perceived attractiveness of a recommended activity (Shah et al., 2023). Furthermore, when influential key opinion leaders highlight the social significance, professional recognition, and public contribution associated with refereeing, audiences are more likely to perceive the profession as socially valuable and personally meaningful.

In addition, key opinion leaders can influence perceptions of cost and convenience value. By systematically presenting information regarding training requirements, certification procedures, promotion pathways, and career opportunities, key opinion leaders reduce information search costs and cognitive effort. Research suggests that greater information accessibility contributes to lower perceptions of behavioral costs and increases perceived decision-making efficiency (Sánchez-Fernández and Jiménez-Castillo, 2021). As effective information intermediaries, key opinion leaders enable potential participants to access and process relevant information more efficiently, thereby strengthening perceptions of convenience and reducing perceived barriers to participation.

Taken together, key opinion leaders contribute to the formation of perceived value through multiple pathways, including the enhancement of functional, emotional, social, cost-related, and convenience-related evaluations. By providing credible information, professional guidance, emotional support, and accessible career knowledge, key opinion leaders increase the overall attractiveness and perceived benefits of soccer referee training and the refereeing profession. Accordingly, KOL characteristics are expected to positively influence women’s perceived value of soccer referee training.

*H3*: KOL characteristics positively influence perceived value among women considering participation in soccer referee training.

In sports research, perceived value has been widely recognized as a key mediating variable in explaining participation intentions, satisfaction, and sustained behavioral engagement. Existing studies suggest that value judgments serve as an important basis for individuals’ behavioral decisions, with higher levels of perceived value significantly promoting the formation of behavioral intentions (Blut et al., 2024). Conversely, when individuals perceive lower value, both their participation intention and their actual participation behavior tend to decline substantially (Wang et al., 2023). Perceived value also represents a comprehensive reflection of individuals’ perceived experiences and psychological benefits associated with a particular profession. Before making behavioral decisions, individuals typically conduct an overall evaluation of the practical benefits and emotional rewards derived from external stimuli and subsequently form behavioral intentions based on these value assessments. In the context of female soccer refereeing, when audiences derive greater satisfaction, emotional motivation, and social recognition from KOL-generated content while simultaneously reducing concerns regarding potential costs, their overall evaluation of the profession becomes more favorable, thereby strengthening their participation intention in referee training. Furthermore, functional, emotional, and social values play important roles in shaping individuals’ career preferences, as people tend to favor professional pathways that provide positive experiences and meaningful benefits. At the same time, improvements in perceived cost value and convenience value can effectively reduce the psychological burden and resource-related concerns associated with career decisions, making individuals more willing to consider entering the profession. As a result, higher perceived value is expected to positively influence female soccer referees’ participation intention in training and may further encourage actual participation behavior.

Although previous studies have highlighted the importance of perceived value in shaping behavioral outcomes, limited research has examined its role within the context of female soccer referee training. Therefore, this study conceptualizes perceived value as a mediating variable in the relationship between KOL characteristics and female soccer referees’ intention and behavior regarding participation in training programs. Based on the above analysis, the following hypotheses are proposed:

*H4*: Perceived value mediates the relationship between KOL characteristics and female soccer referees’ participation intention in training.

*H5*: Perceived value mediates the relationship between KOL characteristics and female soccer referees’ training participation behavior.

#### 2.2.3 The role of perceived fit

Perceived fit reflects an individual’s subjective perception of compatibility and alignment with a particular profession, role, or environment. When individuals form participation intentions or make behavioral decisions, they not only evaluate the value of potential outcomes but also assess the degree to which they perceive themselves as suitable for and compatible with the target profession. In the context of female soccer refereeing, women often encounter multiple barriers, including unfamiliarity with the profession, low self-efficacy, and external skepticism. Furthermore, the role of a soccer referee is characterized by high technical requirements, intense competition, and considerable psychological pressure, all of which may lead potential participants to perceive a relatively low level of fit with the profession.

As a positive external stimulus, the characteristics of Key Opinion Leaders (key opinion leaders) can enhance female audiences’ perceived fit with the refereeing profession through several mechanisms, including role modeling, identity construction, the reduction of cognitive uncertainty, and the visualization of professional competencies (Hsieh, 2023). Through their professional experiences and public visibility, key opinion leaders help audiences better understand the requirements, expectations, and developmental pathways associated with the profession, thereby strengthening perceptions of compatibility and suitability. At present, soccer refereeing is still widely perceived as a male-dominated profession. Women are often constrained by persistent stereotypes and gender-related institutional barriers, which may discourage them from considering refereeing as a viable career option. However, when female key opinion leaders demonstrate professional expertise, participate in high-level competitions, and receive social recognition, they provide visible evidence that women can successfully develop careers within the refereeing profession. Such exposure may strengthen women’s perceptions that female soccer refereeing is both socially accepted and personally attainable (Zvosec et al., 2023). In addition, by communicating industry rules, professional culture, role expectations, and behavioral norms, key opinion leaders help reduce audiences’ sense of unfamiliarity with the profession. This increased transparency enables individuals to gain a clearer understanding of the operational logic and developmental prospects of the refereeing profession, thereby facilitating more positive evaluations of career suitability. Previous research has suggested that greater information transparency enhances individuals’ perceptions of organizational support, professional climate, and future development opportunities (Tenhiälä et al., 2023). Consequently, greater exposure to credible and influential key opinion leaders is likely to strengthen women’s perceived fit with the profession of soccer refereeing. Based on the above analysis, the following hypothesis is proposed:

*H6*: KOL characteristics have a positive impact on perceived fit.

At the same time, existing studies suggest that perceived fit positively influences individuals’ behavioral intentions (Lu R. et al., 2024); Furthermore, the greater the congruence between a KOL’s characteristics and an individual’s self-perceptions, the more likely that individual is to regard the KOL as an important reference when forming behavioral intentions (Belanche et al., 2021). Female soccer referees often face unique psychological pressures arising from gender-related expectations and broader societal influences, particularly within the traditionally male-dominated environment of soccer officiating. In this context, female role models can play an important role in reducing the psychological distance created by gender stereotypes and strengthening women’s confidence in their professional suitability. Consequently, KOL characteristics may not only influence women’s perceptions of the soccer refereeing profession but also enhance their perceived fit with the profession. A higher level of perceived fit can increase motivation among female audiences, which may subsequently influence both their participation intention in referee training and their actual participation behavior (Tal et al., 2024). Furthermore, professional identity theory suggests that when individuals perceive a strong alignment between themselves and a profession, they are more likely to develop a positive professional identity and strengthen their commitment to that profession (Moon et al., 2024). In addition, by sharing professional experiences and career development pathways, key opinion leaders can enhance potential participants’ professional self-efficacy, enabling them to feel more confident about their ability to perform successfully as soccer referees (Midgley et al., 2023). Therefore, perceived fit not only reflects individuals’ evaluations of occupational compatibility but also serves as an important psychological mechanism linking KOL characteristics with participation intentions and subsequent behavioral outcomes. Based on the above analysis, the following hypotheses are proposed:

*H7*: Perceived fit mediates the relationship between KOL characteristics and female soccer referees’ training participation intentions.

*H8*: Perceived fit mediates the relationship between KOL characteristics and female soccer referees’ training participation behavior.

#### 2.2.4 Participation intention in training and participation behavior in training

In the process of individual behavioral decision-making, participation intention is generally regarded as the direct antecedent of behavior and serves as a crucial bridge between cognitive evaluation and actual behavioral engagement. Before engaging in a specific behavior, individuals typically progress through a psychological process that begins with cognitive evaluation, develops into attitudinal formation, and ultimately leads to the establishment of participation intention, which represents the core manifestation of this decision-making process. Existing research has consistently demonstrated that participation intention is a significant predictor of actual behavior. Whether individuals ultimately engage in a particular activity largely depends on the strength of their intrinsic motivation and behavioral propensity. Participation intention also reflects an individual’s level of psychological readiness for behavioral engagement. When participation intention is strong, individuals are more likely to overcome external obstacles and translate their intentions into concrete actions. Conversely, when participation intention is weak, actual participation is less likely to occur, even under favorable external conditions.

This mechanism is particularly evident in the context of female soccer refereeing. Because referee training involves specific professional requirements and practical barriers, individuals typically experience a gradual transition from initial interest to actual participation. Participation intention therefore serves as a critical psychological precursor that facilitates this transition (Downward et al., 2024). Only when individuals develop a strong intention to participate at both the cognitive and emotional levels are they more likely to actively engage in referee training and subsequently translate their intentions into actual participation behavior.

Behavioral intention is regarded as the most direct antecedent of behavior. In the context of female soccer refereeing, where participation in training requires substantial commitment and involves considerable practical challenges, individuals rely more heavily on stable participation intentions to translate motivation into actual behavior. Therefore, the following hypothesis is proposed:

*H9*: Participation intention has a significant positive effect on female soccer referees’ training participation behavior.

Drawing on the literature review and hypothesis development, Figure 1 presents a conceptual model illustrating how KOL characteristics influence female soccer referees’ training participation intentions and behaviors through perceived value and perceived fit.

**Figure 1:**
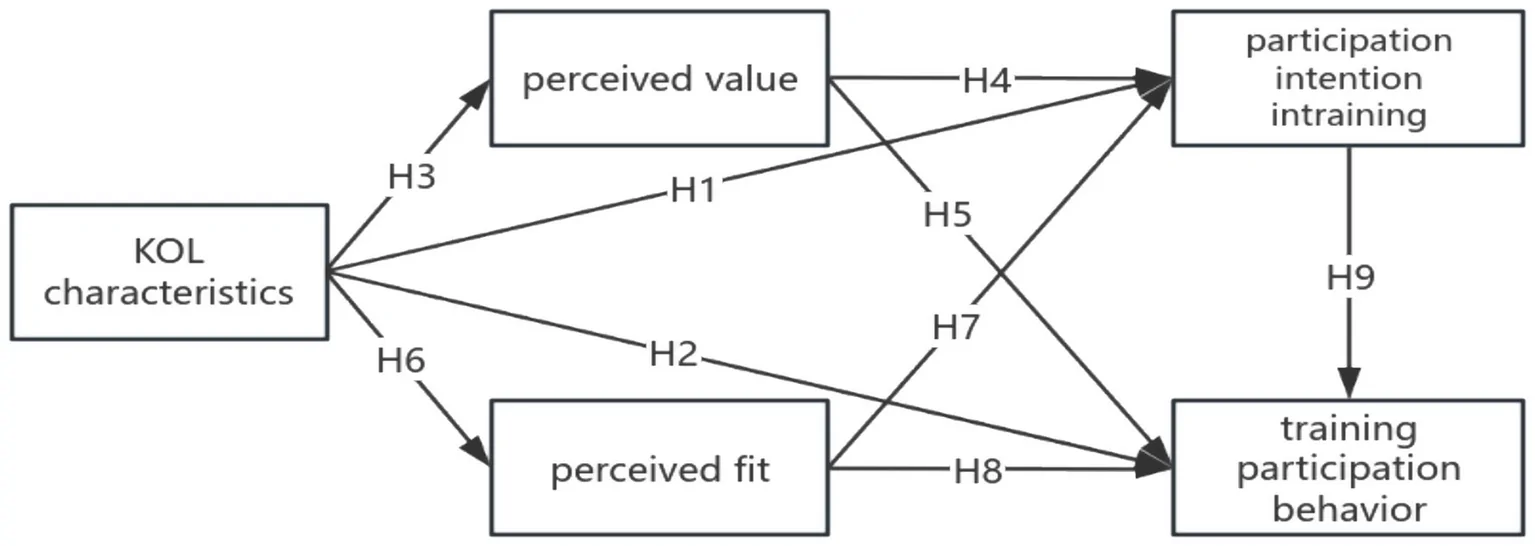
Research model.

## 3 Research design

### 3.1 Research sample

The questionnaire used in this study was developed based on established measurement scales for the relevant constructs reported in previous studies. To ensure its content validity, five professors specializing in physical education were invited to review, discuss, and finalize the questionnaire items. In addition, two native English speakers translated the questionnaire between Chinese and English to ensure linguistic accuracy and consistency.

The study population consisted of adult women who had basic knowledge of or interest in soccer refereeing and had previously participated in soccer-refereeing-related activities. Before completing the questionnaire, participants were informed that only adult women with prior soccer-refereeing-related experience and who held an interest in soccer refereeing were eligible to participate in the survey. The survey was conducted between October and December 2025. Given the geographically dispersed distribution of the target population, data were collected through the online survey platform Wenjuanxing. The questionnaire was distributed through major social media platforms in China. A total of 827 questionnaires were collected from Hubei, Guangdong, and other regions of China. After excluding 83 invalid questionnaires due to careless responses, excessively short completion time, or incomplete information, 744 valid questionnaires were retained for analysis, yielding a valid response rate of 89.96%.

The final sample included respondents from several provinces in China, including Hubei, Guangdong, Liaoning, Jiangsu, and Henan, and exhibited considerable diversity in demographic characteristics, such as age and educational attainment (see Table 1). These characteristics suggest that the sample provides an adequate basis for hypothesis testing in the present study.

**Table 1:** Statistics for the valid sample.

Demographic variables	Category	Frequency	Sample proportion (%)
Level of education	High school and below	31	4.2
	Associate degree	187	25.1
	Undergraduate	420	56.5
	Master’s degree or higher	106	14.2
Age	18–21 years old	282	37.9
	22—25 years old	175	23.5
	26—31 years old	192	25.8
	32 years of age and older	95	12.8
Monthly income	3,000 RMB or less	273	36.7
	3,001–5,000 RMB	232	31.2
	5,001—8,000 RMB	178	23.9
	8,000 RMB or more	61	8.2
Marital Status	Unmarried	524	70.4
	Married with no children	108	14.5
	Married with children	108	14.5
	Other	4	0.5
Frequency of viewing content related to soccer referees	Less than 1 h	247	33.2
	1–3 h	103	13.8
	4–6 h	222	29.8
	More than 6 h	172	23.1

### 3.2 Measurement tools

This study examines five constructs: KOL characteristics, perceived value, perceived fit, participation intention in training, and training participation behavior. All measurement items were assessed using a seven-point Likert scale. The measurement items for each construct are presented in Table 2. KOL characteristics, perceived value, and perceived fit were specified as first-order reflective constructs. Although the measurement items for these constructs covered several conceptually relevant domains, these domains were used to ensure adequate content coverage and were not modeled as separate latent dimensions or as components of second-order constructs.

**Table 2:** Questionnaire items.

Variable	Title	Path load	SD	AVE	CR	KMO
KOL Characteristics	KC1	0.851	1.798	0.658	0.966	0.973
	KC2	0.863	1.809			
	KC3	0.868	1.826			
	KC4	0.877	1.796			
	KC5	0.878	1.792			
	KC6	0.879	1.778			
	KC7	0.879	1.787			
	KC8	0.868	1.786			
	KC9	0.802	1.771			
	KC10	0.86	1.811			
	KC11	0.792	1.738			
	KC12	0.736	1.756			
	KC13	0.658	1.695			
	KC14	0.628	1.658			
	KC15	0.653	1.637			
Perceived value	PV1	0.763	1.642	0.585	0.955	0.947
	PV2	0.77	1.618			
	PV3	0.794	1.645			
	PV4	0.792	1.648			
	PV5	0.804	1.600			
	PV6	0.798	1.602			
	PV7	0.75	1.596			
	PV8	0.732	1.556			
	PV9	0.743	1.565			
	PV10	0.762	1.647			
	PV11	0.784	1.573			
	PV12	0.691	1.604			
	PV13	0.778	1.630			
	PV14	0.741	1.580			
	PV15	0.758	1.588			
Perceived Fit	PF1	0.772	1.598	0.580	0.925	0.910
	PF2	0.751	1.588			
	PF3	0.766	1.646			
	PF4	0.722	1.576			
	PF5	0.742	1.570			
	PF6	0.748	1.550			
	PF7	0.783	1.583			
	PF8	0.785	1.599			
	PF9	0.781	1.591			
participation intention in training	PI1	0.853	1.607	0.725	0.888	0.746
	PI2	0.862	1.609			
	PI3	0.84	1.562			
Training Participation Behavior	PB1	0.875	1.630	0.744	0.897	0.752
	PB2	0.859	1.563			
	PB3	0.853	1.601			

#### 3.2.1 KOL characteristics scale

The measurement of KOL characteristics was based on the scale developed by Ohanian (1990). This scale assesses five dimensions—expertise, popularity, interactivity, homogeneity, and involvement—and requires participants to complete the questionnaire based on their past experiences with sports activities. It consists of 15 items rated on a 7-point scale ranging from 1 (strongly disagree) to 7 (strongly agree); a higher average score indicates a higher degree of key opinion leader characteristics. In this study, the Cronbach’s α coefficient was 0.966 (>0.9), and the KMO statistic was 0.973.

#### 3.2.2 Perceived value scale

The measurement of perceived value was based on the scale developed by Sweeney and Soutar (2001). This scale consists of five dimensions: functional value, emotional value, social value, convenience value, and cost value. The questionnaire comprises 15 items and uses a 7-point rating scale ranging from 1 (strongly disagree) to 7 (strongly agree); a higher score indicates a higher level of perceived value. In this study, the Cronbach’s α coefficient was 0.955 (>0.9), and the KMO statistic was 0.947.

#### 3.2.3 Perceived fit scale

The measurement of perceived fit was based on the scale developed by Kunz et al. (2011). This scale comprises individual fit, social fit, and industry fit, consisting of a total of 9 items rated on a 7-point scale ranging from 1 (strongly disagree) to 7 (strongly agree); higher scores indicate a higher level of perceived fit. In this study, the Cronbach’s α coefficient was 0.925 (>0.9), and the KMO statistic was 0.910.

#### 3.2.4 Participation intention in training

The measurement of participation intention in training was based on the scale developed by Yang (2006). The scale consists of 3 items and uses a 7-point rating scale ranging from 1 (strongly disagree) to 7 (strongly agree); a lower mean score indicates a lower level of participation intention. In this study, the Cronbach’s α coefficient was 0.888 (>0.9), and the KMO coefficient was 0.746.

#### 3.2.5 Training participation behavior

The measurement of training participation behavior was based on a scale revised by Yi and Gong (2013). The scale consists of 3 items and uses a 7-point rating scale ranging from 1 (strongly disagree) to 7 (strongly agree); a lower mean score indicates a lower level of participation behavior. In this study, the Cronbach’s α coefficient was 0.897 (>0.8), and the KMO statistic was 0.752.

### 3.3 Data analysis methods

This study employed a cross-sectional questionnaire survey to investigate the factors influencing women’s participation in soccer referee training. Data were analyzed using SPSS 27.0 and AMOS 31.0. Specifically, SPSS 27.0 was used for data entry, descriptive statistical analysis, correlation analysis, variance inflation factor (VIF) calculation for multicollinearity detection, and Harman’s single-factor test to assess potential common-method variance (CMV). AMOS 31.0 was employed to conduct confirmatory factor analysis (CFA) and structural equation modeling (SEM) and the common latent method factor (CLMF) test as a further evaluation of CMV. In addition, the significance of the parallel mediation effects was evaluated using the Bootstrap method with 5,000 bootstrap resamples.

Hierarchical regression analysis focuses on examining the individual relationships among variables, whereas structural equation modeling (SEM), combined with the Bootstrap method, enables the simultaneous assessment of complex influence pathways through overall model fit and statistical robustness. The combination of these analytical approaches provides complementary evidence for hypothesis testing, thereby enhancing the robustness and reliability of the study’s findings.

## 4 Results and discussion

### 4.1 Test for deviation from the common method

To examine the potential presence of common method bias, this study employed an anonymous questionnaire administration procedure and conducted Harman’s one-factor test as well as a common latent method factor (CLMF) analysis. Specifically, an exploratory factor analysis was performed on all measurement items to assess the extent of common method variance. The results showed that the first unrotated factor accounted for 30.392% of the total variance, which is below the commonly accepted threshold of 40%. In addition, a CLMF was incorporated into the measurement model to further assess potential common method variance. The change in the comparative fit index (CFI) between the baseline measurement model and the CLMF model was 0.003, indicating that the inclusion of the common method factor did not materially improve model fit. Therefore, common method bias is unlikely to be a serious concern in the present study.

### 4.2 Measurement model assessment

Confirmatory factor analysis (CFA) was conducted using AMOS 31.0 to evaluate the measurement model. The results demonstrated a satisfactory model fit, with the following fit indices: *χ*^2^/df = 1.841, RMR = 0.081, GFI = 0.916, AGFI = 0.897, NFI = 0.952, TLI = 0.973, CFI = 0.977, and RMSEA = 0.034. These results indicate that the proposed measurement model achieved an acceptable fit with the observed data. In addition, to distinguish participation intention from participation behavior, this study constructed an alternative model in which participation intention and participation behavior were combined into a single latent variable. This alternative model was compared against the Baseline model where participation intention and participation behavior were treated as separate latent constructs. Both models attained adequate fit levels. The Baseline model produced marginally better fit indices and a lower AIC value, while the more parsimonious Combined intention-behavior model yielded a lower BIC value owing to fewer estimated parameters (see Table 3). The model comparison results indicate that, although participation intention and participation behavior are highly correlated, they are empirically distinguishable constructs and should therefore be retained separately.

**Table 3:** Comparison of fit indices for alternative measurement models.

Model	*χ*^2^/df	GFI	AGFI	NFI	TLI	CFI	RMSEA	AIC	BIC
Baseline	1.841	0.916	0.897	0.952	0.973	0.977	0.034	1936.428	2835.776
Combined	1.859	0.914	0.896	0.95	0.972	0.976	0.034	1949.868	2784.648

As presented in Table 2, all standardized factor loadings exceeded the recommended threshold of 0.60, indicating that each measurement item adequately represented its corresponding construct. Furthermore, the composite reliability (CR) values for all constructs exceeded the recommended threshold of 0.70, demonstrating satisfactory internal consistency reliability. Discriminant validity was further assessed using bias-corrected bootstrap confidence intervals for the latent correlations. The latent correlation between perceived value and perceived fit was 0.795, with a 95% bias-corrected confidence interval of [0.745, 0.841], while the latent correlation between participation intention and training participation behavior was 0.972, with a 95% bias-corrected confidence interval of [0.945, 0.994]. Neither confidence interval included 1.00, providing evidence that the respective constructs were empirically distinguishable.

Overall, the results of the confirmatory factor analysis indicate that the measurement model has acceptable reliability and discriminant validity, provides a sound foundation for subsequent structural model analysis.

### 4.3 Descriptive statistics and correlation analysis

The descriptive statistics for the study variables are presented in Table 3. The mean score for KOL characteristics was 4.28, indicating that respondents generally held favorable perceptions of the KOL characteristics examined in this study. The mean scores for both perceived value and perceived fit were 4.85, suggesting that respondents reported relatively high levels of value perception and perceived compatibility with the profession. In addition, the mean scores for both participation intention in training and actual training participation were 4.70, indicating that respondents demonstrated relatively high levels of both participation intention in training and training participation behavior.

To preliminarily examine the relationships among KOL characteristics, perceived value, perceived fit, participation intention in training, and training participation behavior, Pearson correlation analyses were conducted (see Table 4). The results indicate that KOL characteristics were significantly and positively correlated with perceived value (*r* = 0.533, *p* < 0.01), perceived fit (*r* = 0.419, *p* < 0.01), participation intention in training (*r* = 0.537, *p* < 0.01), and training participation behavior (*r* = 0.533, *p* < 0.01).

**Table 4:** Mean values, standard deviations, and Pearson correlation coefficients for each variable.

Variable	M ± SD	1	2	3	4	5
1. KOL Characteristics	4.28 ± 1.45	1				
2. Perceived value	4.85 ± 1.26	0.533**	1			
3. Perceived Fit	4.85 ± 1.26	0.419**	0.749**	1		
4. Participation intention in training	4.70 ± 1.44	0.537**	0.601**	0.555**	1	
5. Training Participation Behavior	4.70 ± 1.46	0.533**	0.604**	0.557**	0.867**	1

* indicates *p* < 0.05, ** indicates *p* < 0.01, and *** indicates *p* < 0.001; the same notation applies to the figures below. M denotes the mean, and SD denotes the standard deviation.

In addition, perceived value was significantly and positively correlated with perceived fit (*r* = 0.749, *p* < 0.01), participation intention in training (*r* = 0.601, *p* < 0.01), and training participation behavior (*r* = 0.604, *p* < 0.01). Perceived fit was also significantly and positively correlated with participation intention in training (*r* = 0.555, *p* < 0.01) and training participation behavior (*r* = 0.557, *p* < 0.01).

Finally, participation intention in training was significantly and positively correlated with actual training participation behavior (*r* = 0.867, *p* < 0.01). These findings provide preliminary empirical support for the proposed relationships among the study variables and establish a foundation for the subsequent mediation analyses.

### 4.4 Structural model testing

A structural equation model (SEM) was estimated using AMOS 31.0 to examine the hypothesized relationships among the study variables. Prior to testing the structural model, multicollinearity among the predictor variables was assessed using tolerance and variance inflation factor (VIF) statistics. The tolerance values ranged from 0.362 to 0.642, while the VIF values ranged from 1.558 to 2.763. These values were within acceptable ranges, indicating that severe multicollinearity was not evident among the predictor variables. In the model, KOL characteristics were specified as the independent variable, perceived value and perceived fit as the mediating variables, and participation intention in training and training participation behavior as the dependent variables (see Figure 2).

**Figure 2:**
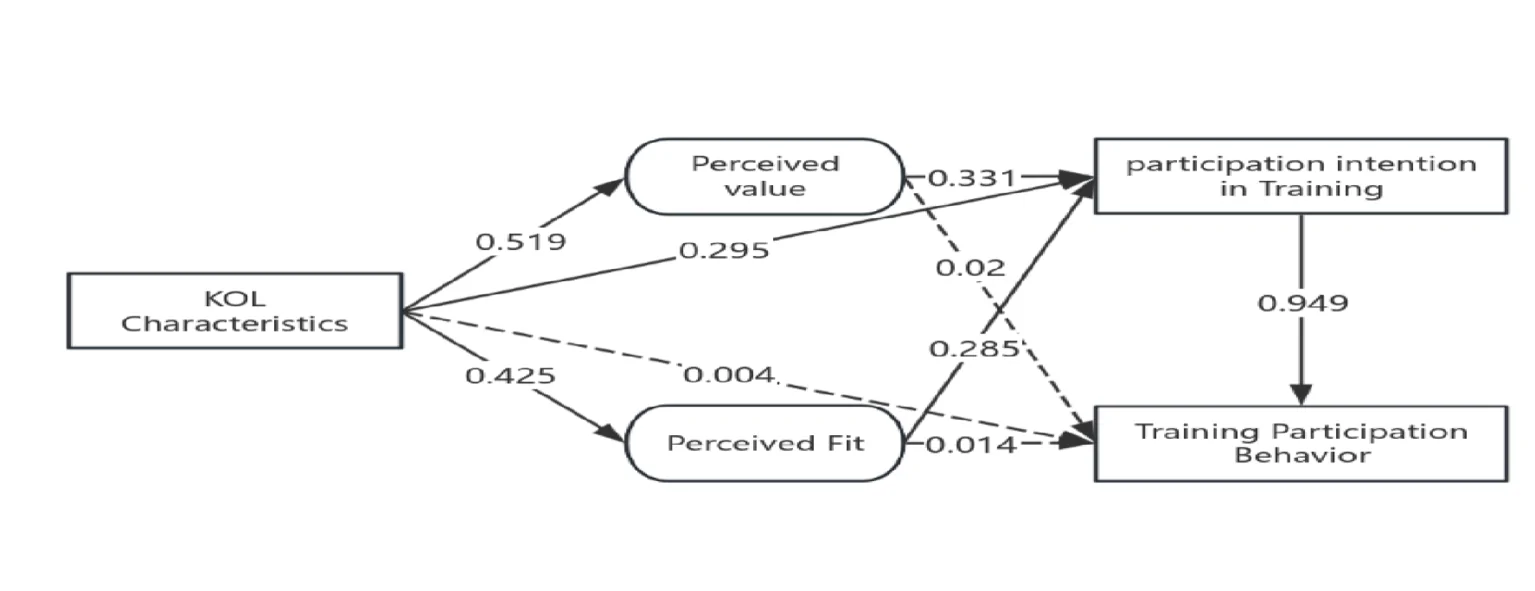
Main path coefficients in the conceptual model. Arrows indicate the direction of the path; solid arrows indicate significant paths, while dashed arrows indicate non-significant paths. *p < 0.05, **p < 0.01, ***p < 0.001.

The model demonstrated a satisfactory fit to the data, with the following fit indices: *χ*^2^/df = 2.889, RMR = 0.386, GFI = 0.851, AGFI = 0.833, NFI = 0.916, TLI = 0.939, CFI = 0.944, and RMSEA = 0.05. These fit indices indicate that the structural model adequately fits the observed data. The standardized path coefficients are presented in Table 5 and Figure 2.

**Table 5:** Regression analysis results.

Path	Standardized estimate	Standard error (S. E.)	Critical ratio (C. R.)	Significance
KOL Characteristics → participation intention in training	0.295	0.035	7.162	*P* < 0.001
KOL Characteristics → Training Participation Behavior	0.004	0.025	0.125	*P* > 0.05
KOL Characteristics → Perceived value	0.519	0.031	13.152	*P* < 0.001
KOL Characteristics → Perceived Fit	0.425	0.031	10.412	*P* < 0.001
Perceived value → participation intention in training	0.331	0.043	8.355	*P* < 0.001
Perceived value → Training Participation Behavior	0.020	0.032	0.724	*P* > 0.05
Perceived Fit → participation intention in training	0.285	0.042	7.396	*P* < 0.001
Perceived Fit → Training Participation Behavior	0.014	0.031	0.501	*P* > 0.05
participation intention in training → Training Participation Behavior	0.949	0.045	21.763	*P* < 0.001

Arrows indicate the direction of the path; solid arrows indicate significant paths, while dashed arrows indicate non-significant paths. *p < 0.05, **p < 0.01, ***p < 0.001.

The results indicate that KOL characteristics had a significant positive structural path on participation intention in training (*β* = 0.295, *p* < 0.001), thereby supporting Hypothesis H1. However, the direct effect of KOL characteristics on training participation behavior was not significant (*β* = 0.004, *p* > 0.05); therefore, Hypothesis H2 was not supported.

The KOL characteristics also had significant positive effects on perceived value (*β* = 0.519, *p* < 0.001) and perceived fit (*β* = 0.425, *p* < 0.001), supporting Hypotheses H3 and H6, respectively.

Furthermore, perceived value (*β* = 0.331, *p* < 0.001) and perceived fit (*β* = 0.285, *p* < 0.001) both exerted significant positive structural influence on participation intention in training, thereby supporting Hypotheses H4 and H7.

By contrast, the effects of perceived value (*β* = 0.020, *p* > 0.05) and perceived fit (*β* = 0.014, *p* > 0.05) on training participation behavior were not statistically significant. Accordingly, Hypotheses H5 and H8 were not supported.

Finally, participation intention in training had a significant and strong positive influence on training participation behavior (*β* = 0.949, *p* < 0.001), supporting Hypothesis H9.

### 4.5 Testing for mediating effects

To further examine the mediating roles of perceived value and perceived fit in the relationship between KOL characteristics and female soccer referees’ participation intention in training and training participation behavior, mediation analyses were conducted using the Bootstrap method. Specifically, 5,000 bootstrap resamples were generated, and bias-corrected 95% confidence intervals (CIs) were estimated. The mediation effect was considered significant when the confidence interval did not include zero.

As shown in Table 6, the indirect effects of the KOL characteristics → perceived value → participation intention in training pathway and the KOL characteristics → perceived fit → participation intention in training pathway were both significant (*p* < 0.001). The standardized indirect effects were 0.172 and 0.121, with corresponding 95% bias-corrected CIs of [0.102, 0.255] and [0.068, 0.195], respectively. The total indirect effect of KOL characteristics on participation intention in training through perceived value and perceived fit was 0.293, with a 95% CI of [0.222, 0.372]. Because the direct effect remained significant, perceived value and perceived fit both showed partial mediating effects.

**Table 6:** Test of mediating effects.

Indirect pathway	Standardized indirect effect	Boot SE	95% BC CI		*p*-value	Hypothesis
			Lower	Upper		
KOL Characteristics → Perceived Value → participation intention in training	0.172	0.039	0.102	0.255	<0.001	H4 supported
KOL Characteristics → Perceived Fit → participation intention in training	0.121	0.032	0.068	0.195	<0.001	H7 supported
KOL Characteristics → Perceived Value → Training Participation Behavior	0.011	0.032	−0.054	0.073	0.742	H5 not supported
KOL Characteristics → Perceived Fit → Training Participation Behavior	0.006	0.019	−0.031	0.046	0.767	H8 not supported
KOL Characteristics → participation intention in training → Training Participation Behavior	0.28	0.046	0.192	0.372	<0.001	Significant indirect path
KOL Characteristics → Perceived Value → participation intention in training → Training Participation Behavior	0.163	0.04	0.094	0.249	<0.001	Significant serial indirect path
KOL Characteristics → Perceived Fit → participation intention in training→ Training Participation Behavior	0.115	0.03	0.066	0.184	<0.001	Significant serial indirect path
Total indirect effect: KOL Characteristics → Participation Intention	0.293	0.038	0.222	0.372	<0.001	Significant
Total indirect effect: KOL Characteristics → Training Participation Behavior	0.574	0.036	0.505	0.644	<0.001	

For Training Participation Behavior, the indirect effects of the KOL characteristics → perceived value → Training Participation Behavior pathway and the KOL characteristics → perceived fit → Training Participation Behavior pathway were not significant. The standardized indirect effects were 0.011 and 0.006, with corresponding 95% CIs of [−0.054, 0.073] and [−0.031, 0.046], respectively. However, the KOL characteristics → participation intention in training → Training Participation Behavior pathway was significant, with an indirect effect of 0.280 and a 95% CI of [0.192, 0.372]. In addition, the serial indirect effect through perceived value and participation intention in training was 0.163, with a 95% CI of [0.094, 0.249], while the serial indirect effect through perceived fit and participation intention in training was 0.115, with a 95% CI of [0.066, 0.184]. Both serial indirect effects were significant.

Overall, perceived value and perceived fit partially mediated the relationship between KOL characteristics and participation intention in training, but did not independently mediate the relationship between KOL characteristics and Training Participation Behavior. Participation intention in training and the serial indirect pathways involving participation intention in training played important roles in the relationship between KOL characteristics and Training Participation Behavior.

## 5 Discussion

### 5.1 The role of KOL characteristics in influencing female soccer referees’ participation intention in training and training participation behavior

The results indicate that, before considering the mediating effects of perceived value and perceived fit, KOL characteristics had a significant positive effect on women’s participation intention in soccer referee training (*β* = 0.295, *p* < 0.001). However, KOL characteristics did not have a significant direct effect on women’s training participation behavior (*β* = 0.004, *p* > 0.05).

According to the Stimulus–Organism–Response (S-O-R) theory, KOL characteristics function as external stimuli that influence women through digital communication environments. As women gradually develop stronger identification with key opinion leaders, their intention to participate in soccer referee training is correspondingly higher. Consequently, KOL characteristics serve as important external cues that shape audience attitudes and behavioral intentions (Hsieh, 2023). Compared with relying solely on personal judgment, women facing the soccer refereeing profession—which is characterized by professional barriers and information asymmetry—are more likely to seek guidance from information sources with professional expertise and social influence. Such information helps reduce cognitive uncertainty and facilitates the formation of stable judgments (Crespo and Tille, 2024).

From the perspective of the underlying mechanism, key opinion leaders provide a valuable reference framework through the sharing of professional experience and behavioral modeling, enabling potential participants to develop a clearer understanding of the nature of the refereeing profession and its career development pathway (Koh et al., 2023). At the same time, the social recognition and value orientation conveyed by key opinion leaders further reinforce individuals’ attitude formation and behavioral tendencies (Lou and Yuan, 2019). In the context of female soccer refereeing, where many women have limited direct exposure to the profession, external authoritative information becomes particularly important in reducing uncertainty and strengthening participation intention.

However, KOL characteristics did not exert a significant direct effect on actual training participation behavior. This finding suggests that observed influence-related for key opinion leaders primarily operates at the cognitive and attitudinal levels and are insufficient, by themself, to directly translate into behavioral. From the perspective of behavioral decision-making, participation in referee training is a high-cost and high-threshold behavior that depends not only on external informational stimuli but also on individuals’ intrinsic motivation and available resources (Rodríguez-Rad and Sánchez del Rio-Vázquez, 2023). Therefore, key opinion leaders primarily facilitate the formation of participation intention rather than directly inducing actual participation behavior. In the context of female soccer refereeing, participation in referee training requires substantial investments of time and effort, including attending training sessions, completing examinations, and engaging in practical officiating activities. Consequently, even when women develop strong participation intentions, these intentions may not immediately translate into actual training participation.

### 5.2 The impact of KOL characteristics on perceived value and perceived fit

The results demonstrate that KOL characteristics have significant positive effects on both perceived value (*β* = 0.519, *p* < 0.001) and perceived fit (*β* = 0.425, *p* < 0.001). These findings suggest that KOL characteristics effectively shape women’s cognitive evaluations of the soccer refereeing profession. This result is consistent with the Stimulus–Organism–Response (S-O-R) theory, which proposes that external stimuli influence individuals’ internal cognitive states, thereby affecting subsequent psychological and behavioral responses.

First, the positive effect of KOL characteristics on perceived value indicates that opinion leaders with high levels of professionalism, credibility, and social influence help women develop a more comprehensive understanding of the career prospects, opportunities for professional development, and social value associated with soccer refereeing. As a result, women are more likely to perceive the profession as valuable and worthwhile (Guest and Cox, 2009). Moreover, the information provided by key opinion leaders reduces uncertainty during the career decision-making process, thereby improving women’s overall evaluation of the soccer refereeing profession (Fisher et al., 1979).

Second, the positive effect of KOL characteristics on perceived fit suggests that key opinion leaders function not only as information providers but also as professional role models. By sharing their professional experiences and career trajectories, female referee key opinion leaders encourage potential participants to engage in self-comparison and role identification, enabling them to perceive a stronger alignment between their own characteristics and the requirements of the refereeing profession. Consequently, women develop greater confidence in their ability to become soccer referees (Morgenroth et al., 2015).

Overall, KOL characteristics influence not only women’s evaluations of the value of pursuing a career in soccer refereeing but also their perceptions of their compatibility with the profession. By simultaneously enhancing perceived value and perceived fit, key opinion leaders establish an important psychological foundation for women’s subsequent participation intention in referee training.

### 5.3 The mediating effect of perceived value

The results indicate that perceived value plays a significant mediating role in the relationship between KOL characteristics and participation intention in training (indirect effect = 0.172, *p* < 0.001), indicating partial mediation. However, the indirect effect of KOL characteristics on Training Participation Behavior through perceived value alone was not statistically significant (indirect effect = 0.011, 95% CI [−0.054, 0.073]). Further analysis showed a significant serial indirect effect through perceived value and participation intention in training (indirect effect = 0.163, 95% CI [0.094, 0.249]).

These findings suggest that KOL characteristics do not directly influence actual training participation behavior but instead correlate with participation decisions indirectly by higher women’s overall perceptions of the value associated with the soccer refereeing profession (Lu H.-H. et al., 2024). When considering career choices, individuals typically evaluate whether pursuing a particular profession is worthwhile before making behavioral decisions (Diegelman and Subich, 2001). Through the sharing of professional experience and the integration of relevant information, key opinion leaders effectively reduce information asymmetry and enable potential participants to develop clearer evaluations of the benefits and value of the refereeing profession (Crespo and Tille, 2024). Consequently, perceived value functions as a key cognitive evaluation mechanism linking external informational stimuli with subsequent participation decisions, highlighting the importance of rational cognitive evaluation in the process of sports career participation.

### 5.4 The mediating effect of perceived fit

The results indicate that perceived fit plays a significant mediating role in the relationship between KOL characteristics and participation intention in training (indirect effect = 0.121, *p* < 0.001), indicating partial mediation. However, the indirect effect of KOL characteristics on Training Participation Behavior through perceived fit alone was not statistically significant (indirect effect = 0.006, 95% CI [−0.031, 0.046]). Further analysis revealed a significant serial indirect effect through perceived fit and participation intention in training (indirect effect = 0.115, 95% CI [0.060, 0.184]).

These findings suggest that women’s decisions to participate in soccer referee training depend not only on evaluations of the value of the profession but also on their perceptions of their compatibility with it (Xu et al., 2023). From a theoretical perspective, this finding can be explained through the mechanisms of social comparison and role identification. By observing the professional performance, career experiences, and behavioral characteristics of opinion leaders, individuals compare these attributes with their own characteristics and gradually develop perceptions of their fit with the refereeing profession (Downes et al., 2021).

In the context of female soccer refereeing, where the profession has traditionally been characterized by gender-related structural barriers, women are more likely to rely on external role models and professional reference points when evaluating their own career suitability (Chhaochharia et al., 2022). Consequently, perceived fit functions as a key psychological mechanism linking KOL characteristics with participation intention in training and is further related to training participation behavior through participation intention in training.

### 5.5 The mediating effects of perceived value and perceived fit

The results demonstrate that perceived value and perceived fit both play significant mediating roles in the relationship between KOL characteristics and participation intention in training. Specifically, KOL characteristics retain a significant direct effect on participation intention in training (*β* = 0.295, *p* < 0.001), while also exerting significant indirect effects through perceived value and perceived fit.

By contrast, neither perceived value nor perceived fit independently mediated the relationship between KOL characteristics and Training Participation Behavior. However, significant indirect effects were observed through participation intention in training and through the sequential pathways involving perceived value or perceived fit together with participation intention in training. These findings suggest that perceived value and perceived fit are more closely related to training participation behavior through their relationships with participation intention in training.

From a mechanistic perspective, key opinion leaders may support individuals’ ability to evaluate the benefits and costs associated with the soccer refereeing profession by integrating information and sharing professional experiences, thereby strengthening perceived value (Crespo and Tille, 2024). At the same time, through professional role modeling and the communication of social recognition, key opinion leaders encourage individuals to engage in self-comparison and role identification, thereby enhancing perceived fit (Spaans et al., 2023). In the context of female soccer refereeing, these two mechanisms, respectively, represent a rational evaluation pathway and a role identification pathway. Together, these mechanisms contribute to the formation of participation intention in training and are further related to Training Participation Behavior through participation intention in training.

### 5.6 The influence of participation intention on participation behavior

The results indicate that participation intention has a significant positive influence on training participation behavior (*β* = 0.949, *p* < 0.001). The substantial path coefficient further suggests that participation intention plays a critical role in translating individuals’ psychological perceptions into actual behavioral engagement. This finding also helps explain why KOL characteristics and the two mediating variables did not exert significant direct effects on training participation behavior. Rather than directly triggering behavioral action, external stimuli influence actual participation through the formation of individuals’ internal participation intention.

This finding aligns with prior work showing that behavioral intention is the most direct antecedent of behavior and is strongly linked to whether individuals are willing to overcome practical constraints and translate their intentions into action. Similarly, previous research has demonstrated that participation intention is a strong predictor of subsequent behavioral engagement (Tsou et al., 2023). In the context of female soccer refereeing, participation in referee training requires substantial investments of time, effort, and professional skill development (McEwan et al., 2023). Consequently, women with stable and strong participation intention are more likely to report sustained training participation behavior.

Overall, participation intention serves as a critical psychological bridge linking external influences and internal psychological perceptions with actual training participation behavior. This finding further supports the S-O-R framework by illustrating that observed behavioral responses are ultimately reflected in the formation of stable participation intentions.

## 6 Theoretical and practical implications

### 6.1 Theoretical implications

First, this study extends the application of the S-O-R theory by providing preliminary evidence of its explanatory relevance in understanding female participation in professional sports development contexts. Previous studies based on the S-O-R framework have primarily examined consumer responses, service experiences, and online behavioral intentions, emphasizing how external stimuli influence individual responses through internal psychological states (Eroglu et al., 2001). However, relatively limited attention has been paid to how environmental and social stimuli are related to individuals’ career-related participation decisions in sports. By identifying KOL characteristics as external stimuli (S), perceived value and perceived fit as internal psychological states (O), and training participation intention and behavior as behavioral responses (R), this study extends the application of the theoretical boundary of the S-O-R framework from consumption-related behaviors to professional sports participation.

Furthermore, previous research on female soccer referees has predominantly focused on structural constraints, gender inequality, and organizational barriers (Cosentino et al., 2021; Drury et al., 2022), while the cognitive processes through which women evaluate and decide to enter referee training remain insufficiently understood. This study addresses this gap by showing that external professional influences were significantly related to internal cognitive evaluations and participation outcomes, while their direct relationship with training participation behavior was not significant. These findings provide a more refined explanation of how female soccer referees’ participation decisions are psychologically constructed.

Second, this study contributes to extending KOL theory beyond its traditional applications in consumer behavior and marketing communication. Previous studies have primarily conceptualized key opinion leaders as influential information sources who shape consumers’ attitudes, perceptions, and purchase intentions through credibility, expertise, and social influence (Lou and Yuan, 2019; Sokolova and Kefi, 2020). However, whether and how key opinion leaders influence individuals’ professional development decisions remains underexplored.

By applying KOL theory to the context of female soccer refereeing, this study suggests that key opinion leaders can function not only as information providers but also as professional role models who influence occupational perceptions and career-related evaluations. Specifically, the findings indicate that KOL characteristics are positively related to women’s perceptions of the value and suitability of referee training, as well as their willingness to participate. This suggests a broader theoretical role of key opinion leaders from influencing short-term consumption decisions to facilitating long-term professional participation decisions.

In addition, this study responds to previous calls for expanding influencer research beyond commercial contexts by providing preliminary evidence of the social and developmental functions of key opinion leaders in professional identity formation and career exploration.

Third, this study advances understanding of career participation decision-making by identifying the parallel mediating roles of perceived value and perceived fit. Previous research has suggested that individuals’ behavioral decisions are influenced by evaluations of both expected benefits and personal compatibility (Cable and DeRue, 2002; Eccles and Wigfield, 2002). However, limited research has examined how these two cognitive evaluations jointly explain women’s participation in traditionally male-dominated sports occupations.

The findings suggest that women’s decisions to participate in soccer referee training involve two distinct but complementary psychological evaluations: first, whether referee training is valuable and worthwhile; second, whether they perceive themselves as suitable for the referee profession. Perceived value reflects individuals’ judgments regarding the benefits and importance of participation, whereas perceived fit reflects the perceived alignment between personal characteristics and occupational requirements.

By integrating these two mechanisms into the S-O-R framework, this study provides a more comprehensive perspective on the relationships underlying female soccer referees’ participation decisions and enriches existing theories of career choice and professional engagement.

### 6.2 Practical implications

This study provides potential practical implications for increasing women’s participation in soccer referee training, optimizing the talent development system for female soccer referees, and promoting the sustainable development of women’s soccer. First, the findings indicate that the characteristics of key opinion leaders are significantly related to women’s perceptions and participation intention in referee training. Therefore, organizations responsible for referee development may consider leveraging the exemplary roles of outstanding referees, referee instructors, and other influential professionals within the refereeing community. Through experience sharing, professional demonstrations, and the dissemination of successful career cases, such approaches may help strengthen women’s understanding of and trust in the refereeing profession and may contribute to attracting more women to referee training.

Second, this study shows that perceived value and perceived fit are important mediating variables influencing women’s participation intention in referee training. This finding suggests that future initiatives aimed at cultivating female soccer referees may consider placing greater emphasis on women’s psychological identification with and perceived compatibility with the profession. On the one hand, greater attention to communicating professional development opportunities and the potential value of referee training may help strengthen perceived value. On the other hand, providing realistic professional information and role-model experiences may help women evaluate their perceived fit with the refereeing profession, thereby potentially strengthening participation in referee training.

Finally, this study shows a strong positive relationship between participation intention in training and training participation behavior. Therefore, future referee development policies may consider measures that facilitate women’s continued participation once participation intentions have been formed. More systematic practical training opportunities and behavioral support mechanisms, such as improving training arrangements and expanding opportunities for practical officiating.

Collectively, these measures may encourage sustained participation in referee training and may support the long-term development of women’s soccer and the female soccer referee workforce in China.

## 7 Limitations and future directions

Although this study provides empirical evidence for understanding how KOL characteristics influence women’s participation in soccer referee training through perceived value and perceived fit, several limitations should be acknowledged.

First, the generalizability of the findings is limited by the sample and research design. The data were collected from female participants in China using a cross-sectional survey, which may restrict the applicability of the findings across different cultural contexts, regions, and stages of referee development. Participants within the sample may have varying degrees of refereeing or training-related experience; thus, the findings cannot be simply generalized. Moreover, the cross-sectional design limits the ability to capture the dynamic process through which participation intention develops into sustained training behavior. Future research could employ longitudinal designs, cross-cultural comparisons, and mixed-method approaches to further examine the temporal and contextual mechanisms underlying female soccer referees’ participation development.

Second, although this study identifies KOL characteristics, perceived value, and perceived fit as important psychological mechanisms influencing training participation, women’s engagement in soccer refereeing may also be shaped by broader social and institutional factors. Future studies could extend the current framework by incorporating variables such as organizational support, social norms, self-efficacy, and career development environments. Integrating individual-level psychological mechanisms with macro-level contextual factors may provide a more comprehensive understanding of the sustainable development of female soccer referees.

## Data Availability

The raw data supporting the conclusions of this article will be made available by the authors, without undue reservation.
